# Virtual reality locomotion methods differentially affect spatial orientation and cybersickness during maze navigation

**DOI:** 10.1038/s41598-025-12143-y

**Published:** 2025-07-19

**Authors:** Petr Hořejší, Alena Lochmannová, Vojtěch Jezl, Matěj Dvořák

**Affiliations:** 1 Department of Industrial Engineering and Management, Faculty of Mechanical Engineering, University of West Bohemia, Univerzitní 22, Pilsen, 30100 Czech Republic; 2 XR Institute, s.r.o. (Extended Reality Institute), Koterovská 2827/152, Pilsen, 32600 Czech Republic

**Keywords:** Virtual reality, Locomotion methods, Spatial navigation, Cybersickness, User experience, Psychology, Engineering

## Abstract

Virtual reality (VR) is widely used in training, simulations, and industrial applications, yet effective locomotion remains challenging due to its impact on spatial orientation and cybersickness. This study investigates the effects of three locomotion methods—hand-tracking (HTR) with teleportation, traditional VR controllers (CTR), and the mechanical interface Cybershoes (CBS)—on navigation performance, perceived usability, and cybersickness during navigation tasks in virtual mazes of three increasing difficulty levels. The experiment involved 15 participants (M = 22.6 years, SD = 1.64), performing a total of 9 trials each (3 methods × 3 mazes), resulting in 135 exposures overall. The HTR method had the longest average maze completion time (127 ± 54 s for the simplest maze), significantly longer compared to both CTR (52 ± 25 s, *p* < 0.01) and CBS (52 ± 22 s, *p* < 0.01). CBS showed comparable navigation performance to CTR, slightly outperforming CTR only in the most difficult mazes (108 ± 51 s vs. 115 ± 42 s, *p* < 0.05). Regarding usability, CTR received the highest ratings (SUS: 74.67 ± 18.52), followed by CBS (67.83 ± 24.07) and HTR (65.83 ± 22.22). However, CBS induced the highest cybersickness (2.9 ± 1.2), significantly higher than HTR (1.8 ± 0.9; *p* = 0.006), while CTR scored intermediate (2.3 ± 1.1). Results confirm that teleportation (HTR) minimizes cybersickness but negatively impacts spatial orientation. CBS support more efficient navigation in complex tasks but considerably increases cybersickness. Joystick locomotion (CTR) provides the best balance among navigation efficiency, usability, and user comfort. These findings contribute to optimizing locomotion strategies in VR applications.

## Introduction

Virtual reality (VR) has undergone significant technological advancements in recent years, extending beyond the entertainment industry into fields such as scientific research, medicine^1,2^and industrial applications^3^. Recent work has also shown that VR is a promising platform for investigating dual-task cognitive load and visually guided multitasking, with three-dimensional stimulus formats imposing measurably higher demands than their two-dimensional counterparts^4,5^. The ability to provide high levels of immersion and interaction with synthetic environments has established VR as a powerful tool for studying human behavior, cognitive processes, and spatial orientation. Ensuring natural, intuitive, and comfortable locomotion remains a fundamental challenge in VR system design. Locomotion interfaces, which enable users to navigate and alter their direction within virtual environments, represent a critical component of effective human–VR interactions. Their usability is paramount, as it directly influences navigation efficiency, spatial awareness, and the overall quality of user experience. The physiological and psychological demands associated with different locomotion techniques can significantly impact user performance, navigation efficiency, and overall experience^6–8^. VR locomotion often induces sensory conflicts that contribute to cybersickness, a motion-induced discomfort akin to simulator sickness, which remains a major limiting factor in VR adoption^9^. Building on Sensory Conflict Theory, cybersickness occurs when visual input signals movement that is not matched by vestibular or proprioceptive feedback, creating a neural mismatch that the brain struggles to resolve^10,11^. Teleportation (HTR) minimises this conflict by removing continuous optic flow, whereas joystick-based steering (CTR) preserves strong visual acceleration cues without matching vestibular input, often provoking cybersickness. Foot-based devices such as Cybershoes add proprioceptive feedback, which may attenuate—but not eliminate—the mismatch because users remain seated and experience no real linear acceleration, a residual conflict that likely explains the higher discomfort we observed for CBS^12^. This theoretical lens helps account for the distinct comfort profiles of the three locomotion modalities examined in the present study.

Efficient spatial orientation within virtual environments relies on the integration of multimodal sensory inputs^13^including visual, vestibular, and proprioceptive cues. Discrepancies between real-world and virtual sensory feedback frequently lead to distortions in distance estimation, reduced navigational accuracy, and disruptions in cognitive map formation. Spatial orientation is a fundamental cognitive function underpinning movement, navigation, and spatial awareness across daily activities, athletic performance, and complex wayfinding tasks. Spatial navigation enables individuals to maintain directional awareness during movement. Visual-spatial processing facilitates the construction of cognitive maps, which are essential for precise orientation^14,15^. Wayfinding is a higher-order cognitive process involving route learning and memory-based navigation. It also includes evaluating spatial relationships between objects, individuals, and landmarks. The ability to construct and utilize cognitive maps is critical for successful wayfinding and navigation, particularly within immersive virtual environments where natural spatial cues may be altered or absent^16,15^. Despite advancements in VR hardware, persistent challenges—including system latency, constrained haptic feedback, and sensory conflicts—continue to exacerbate cybersickness, negatively impacting user experience and limiting prolonged immersion.

Locomotion techniques in VR constitute a fundamental determinant of user comfort, spatial cognition, and navigation efficiency. Existing locomotion paradigms can be classified into three primary categories: (1) CTR-based movement, which employs joysticks or trackpads to facilitate precise directional control, albeit with limited proprioceptive feedback and physical engagement^17^, (2) HTR systems, which enhance natural interaction with virtual objects but present challenges in executing seamless and continuous movement^18,19^, and (3) mechanical locomotion interfaces, including treadmills and foot-based devices such as CBS, designed to approximate natural gait dynamics while mitigating sensory conflicts^12^. Each modality exhibits distinct advantages and constraints concerning immersion, usability, and susceptibility to cybersickness. Given the critical role of locomotion in shaping user experience and task performance in VR, further empirical investigation is required to refine these techniques and develop optimized locomotion frameworks that balance realism, cognitive load, and user comfort. This study systematically evaluates the impact of different VR locomotion methods on spatial navigation and user experience. Specifically, it compares CTR-based movement, HTR with teleportation, and CBS-assisted navigation in terms of maze completion time, cybersickness severity, and usability. By examining the cognitive and physiological effects of these techniques, the research aims to refine VR locomotion paradigms, enhancing their applicability in training simulations, virtual prototyping, and immersive environments.

### Related work

The influence of VR locomotion techniques on spatial orientation, cognitive load, and user experience has been extensively examined in prior research. Studies indicate that the selection of locomotion modality significantly affects navigational accuracy, task efficiency, and the perceived sense of presence within virtual environments^20^.

Comparative analyses of CTR-based locomotion and physically active techniques, such as walking-in-place (WIP), have demonstrated that the latter enhances spatial memory and route-learning efficiency. Users using WIP show superior recall of spatial layouts and object locations compared to those utilizing joystick-based movement. This effect is attributed to the greater sensorimotor congruence between visual and proprioceptive feedback, which supports the formation of more robust cognitive maps. Jerald (2015)^21^ emphasizes that WIP is a viable alternative in environments with limited tracking space and safety constraints. Despite these potential benefits, involuntary positional shifts (IPS) have been observed with this method, which may compromise navigation stability and spatial accuracy^20^. Similarly, HTR systems, which facilitate natural gestural interaction with virtual environments, have been found to enhance immersion^19,22^. However, they introduce limitations in precise movement execution, often reducing navigation speed and efficiency, depending on factors such as optimization algorithms or interface design. Recent research^23^ has also highlighted that HTR interactions provide rich biometric data, allowing implicit user identification based on movement patterns and interaction styles. Liebers et al. (2024)^23^ demonstrated that individual differences in HTR behaviors, particularly during bimanual interactions, can be used to identify users with up to 95% accuracy in VR environments. These findings suggest that while HTR enhances user immersion, it also introduces variability in navigation efficiency due to unique user-specific motor behaviors.

Teleportation-based locomotion is a widely employed technique for mitigating cybersickness, as it eliminates continuous optical flow and minimizes vestibular-visual conflicts through instantaneous repositioning within the virtual environment. While teleportation effectively reduces motion-induced discomfort, research has identified its potential drawbacks in spatial cognition^24,25^. Users relying on teleportation often exhibit difficulties in accurate distance estimation and are more prone to disorientation compared to those utilizing continuous locomotion methods. Although teleportation has been associated with shorter task completion times, this efficiency gain comes at the expense of reduced spatial presence and impaired awareness of traveled trajectories, which can negatively impact cognitive map formation and spatial orientation^26^.

Studies have also demonstrated that teleportation is among the most comfortable locomotion techniques, with Bozgeyikli et al. (2016)^27^ identifying it, alongside joystick-based movement, as the most preferred options, particularly among individuals with autism spectrum disorder. These techniques were favored for minimizing discomfort and enhancing spatial orientation, suggesting that selecting appropriate locomotion methods can significantly improve user experience and navigation efficiency for specific groups. Research has established a link between locomotion methods and cybersickness. Kumar et al. (2023)^28^ explored how different body postures (standing vs. sitting) and locomotion techniques (joystick vs. teleportation) affect postural stability, cybersickness, and the sense of presence in virtual reality environments. Their findings revealed that the greatest postural instability occurred with joystick use while standing. Conversely, cybersickness was minimized by using teleportation while seated. The sense of presence was highest when teleportation was used in a standing position, suggesting that the optimal combination of locomotion and posture depends on the specific requirements of the VR application. Similarly, Clifton and Palmisano (2020)^29^ compared controlled locomotion and teleportation in VR in terms of cybersickness, sense of presence, and perceived motion. They found that controlled locomotion generally induced more cybersickness than teleportation, although some participants in the teleportation group experienced worse symptoms. The sense of presence gradually increased with controlled locomotion, while it remained stable with teleportation. Langbehn et al. (2018)^30^ further reported that users tend to favor teleportation over joystick-based locomotion due to its association with a lower incidence of motion sickness. Similarly, Buttussi and Chittaro (2021)^31^ found that teleportation not only reduced nausea but also outperformed joystick and leaning locomotion techniques in terms of usability and overall user experience. Kazemi, Kumar, and Lee (2024)^6^ further highlight that teleportation imposes a higher cognitive workload compared to joystick-based locomotion, as indicated by increased theta-band activity in EEG recordings. Their findings suggest that the discontinuous nature of teleportation requires greater cognitive effort, leading to increased task demands, as measured by the NASA Task Load Index (NASA-TLX). Their study demonstrated that standing users experience even higher cognitive load than seated users when utilizing teleportation, underscoring the interaction between locomotion modality, posture, and cognitive strain in VR navigation.

In response to the demand for greater mobility and cost efficiency, recent advancements have introduced wireless wearable systems that utilize foot movement tracking for locomotion control. These systems enable users to navigate virtual environments through natural walking or foot sliding movements, accompanied by body tilting, thereby maintaining an intuitive and immersive experience without the need for stationary hardware. According to Lu and Mao (2021)^32^ such interaction methods, which integrate foot movement tracking with body tilting, help mitigate cybersickness by reducing the discord between visual motion cues and the user’s physical sensory inputs. The effectiveness of these systems is largely contingent on the precise calibration of the device’s speed and sensitivity to bodily movements, which plays a crucial role in enhancing user comfort and the overall naturalness of the interaction. A study by Zhang and Billah^12^ further underscores both the potential and limitations of CBS as a seated-WIP locomotion solution. Their findings indicate that participants generally perceived CBS as a more natural navigation method compared to handheld CTRs, with the majority reporting a reduction in motion sickness. However, challenges such as perceived slower movement speed, ergonomic constraints, and limitations in action detection were also identified. Moreover, their research highlights the applicability of CBS beyond gaming, suggesting potential uses in exercise, professional training, remote work, and accessibility, thereby expanding the scope of VR locomotion interfaces into practical and rehabilitative domains.

Many studies^33^ indicate that the severity of cybersickness is closely linked to factors such as locomotion speed, field of view, duration of exposure, and individual susceptibility. Differences in adaptation rates across locomotion interfaces have also been observed^34^. Sensory conflict, particularly visual-vestibular discrepancies, is recognized as a primary cause of cybersickness^35^. Illusions of self-motion in VR have been linked to its onset, with sensory conflict theories suggesting that steering locomotion induces greater visual-vestibular discord than teleportation^29^. Findings from the study by Kim, Lee, and Lee^34^ support this notion, demonstrating that steering locomotion enhances self-motion perception more than teleportation, thereby intensifying cybersickness symptoms.

While head-mounted display (HMD)-based VR is known for eliciting strong feelings of presence and immersion, it is also a common trigger for motion sickness. Clifton and Palmisano^29^ investigated the relationship between presence, cybersickness, and vection (self-motion perception) across different locomotion techniques, specifically comparing steering locomotion with teleportation. Their findings suggest that although cybersickness occurs in HMD-based VR, it does not significantly diminish the sense of presence. This may be attributed to the fact that HMDs generate a stronger feeling of presence than other VR and simulation modalities, as highlighted by Mondellini et al. (2018)^36^. Understanding the underlying causes of cybersickness is therefore essential for developing effective mitigation strategies, allowing for improved locomotion design without compromising immersion.

To provide a clearer overview of the practical relevance of the three VR locomotion methods studied—hand-tracking teleportation (HTR), controller-based movement (CTR), and Cybershoes (CBS)—Table 1 shortly summarizes their control mechanisms, core advantages and limitations, and real-world use cases.

**Table 1:** Summary of current use, advantages, and limitations of three VR locomotion methods (HTR, CTR, CBS) in research and Applications.

Method	Control Mechanism	Advantages	Limitations	Example Use Cases	Key References
**HTR** (Hand Tracking + Teleportation)	Hand gestures with point-to-teleport mechanics	Minimizes cybersickness; intuitive; no controllers needed	Impaired spatial orientation; slow navigation; limited flow continuity	Gesture-based interaction research; VRChat and exploration modules, VR rehabilitation	( Bozgeyikli et al., 2016 )^27^, ( Kazemi et al., 2024 )^7^
**CTR** (VR Controller/Joystick)	Thumbstick-based steering and rotation	Fast and precise movement; high usability; widely familiar	Moderate cybersickness over time; limited physical engagement	Games (e.g., *Half-Life: Alyx*,* Beat Saber*); industrial training and simulation	( Clifton & Palmisano, 2020 )^29^, ( Langbehn et al., 2018 )^30^
**CBS** (Cybershoes)	Seated or semi-seated foot-sliding mapped to forward movement	Higher proprioceptive realism; supports embodied navigation; good for spatial mapping	Highest cybersickness; sensitive to calibration and seated posture effects	VR rehabilitation, walking-in-place research, training simulations	( Zhang & Billah, 2024 )^12^

### Objectives of the study

Previous research has extensively explored individual locomotion techniques in virtual reality (VR), focusing predominantly on joystick-based navigation and teleportation methods. However, comprehensive comparisons involving emerging mechanical locomotion interfaces—such as Cybershoes (CBS), which combine natural foot movements with body tilting—remain limited. Given that each locomotion method presents distinct trade-offs between navigation efficiency, spatial orientation accuracy, and cybersickness, there is a clear need to systematically evaluate and compare these approaches. The present study addresses this research gap by empirically comparing three VR locomotion paradigms: hand-tracking with teleportation (HTR), traditional joystick controllers (CTR), and CBS-assisted navigation. Results from this study will provide practical insights into optimizing VR locomotion strategies for improved user experience, performance efficiency, and reduced cybersickness in immersive environments.

The research is structured around two central research questions. The first research question (RQ1) seeks to determine the extent to which the average completion time of virtual mazes varies based on the locomotion modality employed, specifically comparing HTR, VR CTRs, and CBS. Hypothesis 1 posits that there are statistically significant differences in maze completion times across these locomotion techniques. In other words, the locomotion modality will have an effect on the time required to complete the maze. Additionally, Hypothesis 1a suggests that the influence of the locomotion modality on completion time will be more pronounced for more complex mazes, with higher complexity increasing the differences in performance across locomotion methods.

The second research question (RQ2) explores whether a relationship exists between navigation speed and subjective user perceptions, specifically (a) the severity of cybersickness symptoms and (b) the perceived usability of the locomotion method, as quantified by the System Usability Scale (SUS). Hypothesis 2 proposes that different locomotion interfaces (HTR, VR CTRs, and CBS) elicit statistically significant differences in cybersickness intensity, with each locomotion method inducing varying degrees of cybersickness. Hypothesis 2a suggests that higher navigation efficiency correlates with higher usability ratings and lower cybersickness severity, with this relationship being stronger for more complex mazes.

By addressing these questions and empirically testing associated hypotheses, this study aims to clarify how different VR locomotion strategies affect navigation efficiency and user comfort. The findings are expected to contribute to optimizing VR interaction paradigms for applications in training simulations, virtual prototyping, and immersive entertainment.

## Methods

This study employed an experimental within-subjects design to evaluate the impact of different virtual reality locomotion methods on spatial orientation and cybersickness.

### Experimental apparatus

**Fig. 1:**
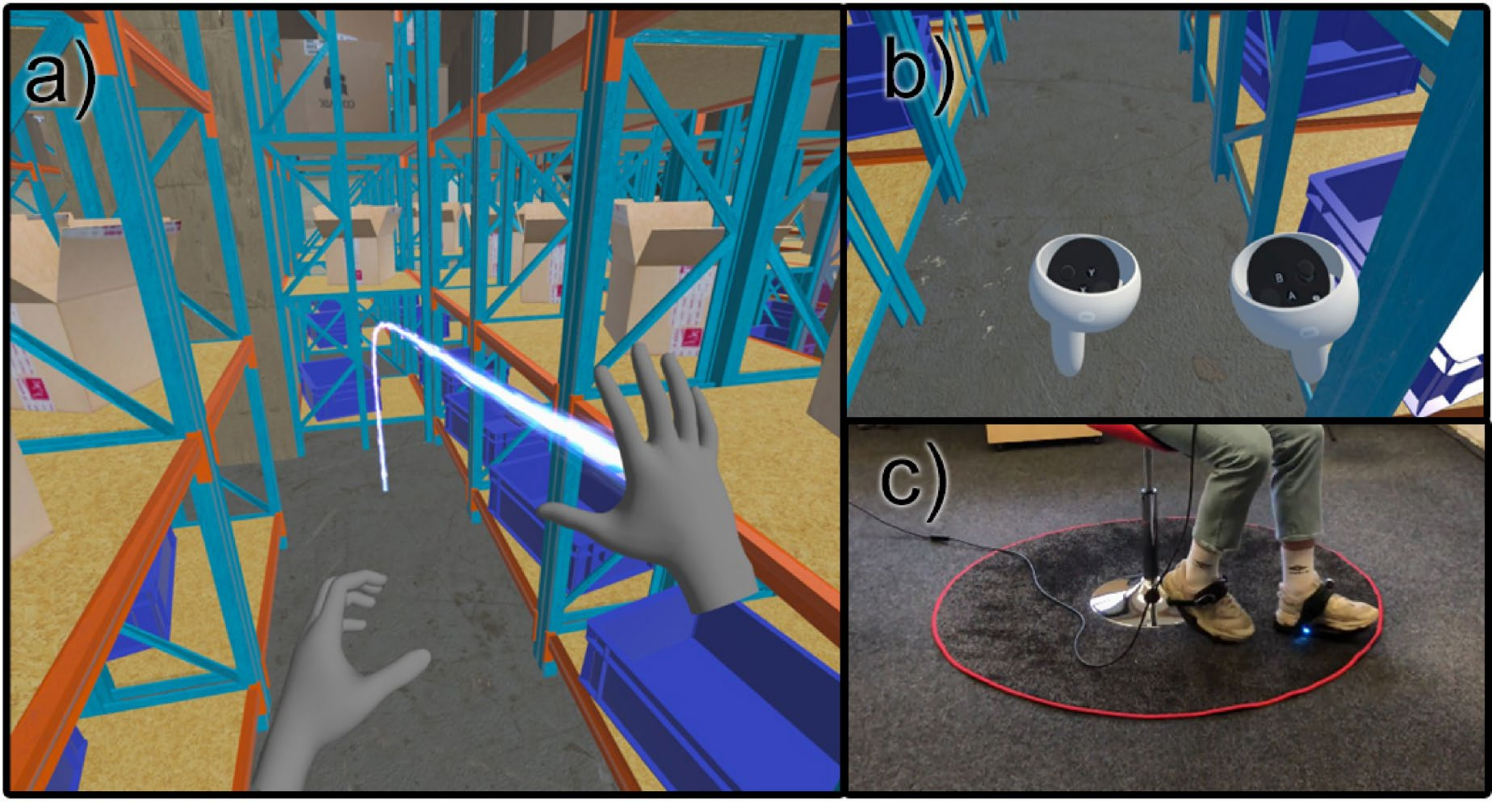
(**a**) HTR with teleportation; (**b**) traditional VR CTRs; (**c**) With the control using CBS, the user does not see any CTRs, as they are not needed in this case. Movement occurs by sliding the feet on the carpet, and rotation is controlled by the user turning in the chair.

The experimental setup was designed to assess spatial orientation and cybersickness across three distinct virtual reality locomotion modalities: HTR with teleportation, traditional VR CTRs, and CBS (see Fig. 1). The first HTR method utilized HTR sensors, allowing users to interact with the environment through natural gestures such as pulling or simulated walking movements. Teleportation through HTR provided a precise and intuitive way to select destinations without adding significant cognitive load, thus enhancing immersion. The second CTR approach relied on CTR-based navigation, where participants used a standard VR joystick to control movement direction and speed. This method provided precise control with smooth translation in the virtual space and allowed for incremental 45-degree rotations, maintaining consistency with traditional gaming locomotion mechanics. The third CBS technique involved CBS-assisted locomotion, where participants, while seated, performed stepping motions to propel themselves forward in the virtual environment. Foot-mounted rotational sensors converted stepping motions into forward displacement. A swivel chair enabled participants to rotate their bodies, aligning real and virtual movements naturally. This method engaged lower-limb proprioception while maintaining a stable seated posture to reduce excessive vestibular conflict. Each locomotion approach was fully integrated into the VR system, providing real-time motion tracking with minimal latency. The effectiveness of these techniques was assessed through a combination of objective performance metrics and subjective user feedback. After each exposure and completion of the questionnaire, participants were also asked follow-up questions to gather further insights into their experiences and feelings regarding each locomotion method. These free-text responses provided additional qualitative data on participants’ subjective experiences.

The VR system utilized two types of head-mounted displays (HMDs) depending on the experimental conditions. For the HTR and CTR variants, a standalone Meta Quest 2 HMD was used, featuring a resolution of 1832 × 1920 pixels per eye and a refresh rate of 72 Hz. For the CBS variant, the Valve Index headset was employed, providing a resolution of 1440 × 1600 pixels per eye and a refresh rate of up to 144 Hz, chosen specifically for compatibility with CBS. The refresh-rate settings reflected the manufacturer-recommended defaults for each device in the software configurations used during data collection. Although the Quest 2 can be over-clocked to 90–120 Hz, those modes were not yet officially supported for untethered applications under the firmware version used; the native 72 Hz profile was therefore retained to ensure stable standalone performance. By contrast, the Valve Index is conventionally operated at 120–144 Hz when tethered to a gaming-class PC, and we observed that lowering the frame rate introduced missed-frame stutter when the Cybershoes driver was polled at high frequency. We therefore kept the default 144 Hz setting to minimise input latency for CBS trials. While higher refresh rates can mitigate cybersickness by reducing display latency^37^, we note this hardware difference as a potential confound and revisit it in the Limitations section. The virtual environment consisted of a series of mazes created in Unity (version 2021.3 LTS), deployed either as a standalone application for the all-in-one Quest 2 headset or operated in a tethered desktop mode when using the Valve Index. Before each session, the HMD was individually calibrated following the respective manufacturer’s guidelines. Lens spacing (inter-pupillary distance) was adjusted with the Quest 2 three-position slider or the Index mechanical IPD dial while a high-contrast test grid was displayed to verify image sharpness across the field of view. Head-strap tension was set so that the display rested evenly on the face without light leakage, and the software guardian boundary was re-defined for each participant. Display brightness and render scale were left at factory defaults to keep colour and resolution consistent across participants. Each headset was operated at its manufacturer-recommended refresh rate and resolution—72 Hz at 1832 × 1920 px per eye for the Quest 2 and 144 Hz at 1440 × 1600 px per eye for the Valve Index—to ensure optimal display stability and low latency. These differences in display quality and latency may have influenced both navigation performance and cybersickness, particularly by enhancing optic-flow fidelity at higher frame rates.

### Maze design and development

The virtual maze environment was constructed to standardize the navigation challenges across all experimental conditions while maintaining a high level of ecological validity. The design was informed by established research on spatial navigation in virtual environments^38^aiming to create an immersive experience by balancing complexity, spatial orientation cues, and locomotion demands. Maze layouts included dead ends and multiple branching routes. These features increased navigational difficulty, ensuring participants faced varied and challenging tasks. These elements were strategically placed to support spatial orientation, with key landmarks such as unique textures and lighting variations.

The maze structure design followed a grid-based system, where each unit represented a potential corridor or wall segment. The layout consisted of multiple interconnected pathways with varying levels of complexity. The optimal path was algorithmically determined, incorporating variations in path complexity across different experimental conditions to ensure fairness while maintaining the challenge for participants. This approach is consistent with the principles of cognitive load and spatial navigation as outlined in studies^39^which demonstrated the impact of maze structure and overlap on disorientation in VR environments.

To maintain consistency while allowing for variability in navigation tasks, a procedural maze generation algorithm was employed, as procedural generation techniques have been shown to effectively create varied and ecologically valid VR environments^40^. This algorithm dynamically generated walls and pathways using a depth-first search (DFS) and recursive backtracking method. Key adjustable parameters, such as corridor length, branching factor, and the number of decision points, ensured that the mazes maintained a controlled yet unpredictable layout. Each generated maze adhered to predefined difficulty constraints, preventing overly simplistic or overly convoluted structures that could skew the experimental outcomes.

**Fig. 2:**
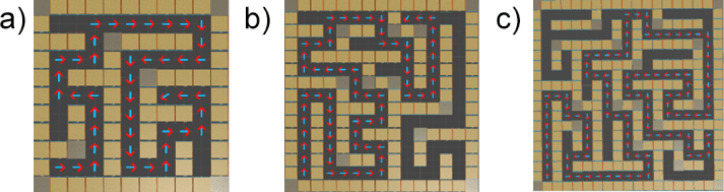
Example of one maze design variant (**a**) Easy (**b**) Moderate (**c**) Hard.

The different difficulty levels of the maze (see Fig. 2) were determined based on the quantification of several key parameters, including the size of the maze (number of cells), the number of steps required to reach the goal, the number of dead ends, and the total length of the optimal path to the goal. The low difficulty level was represented by a maze of 11 × 11 cells, requiring a maximum of 15 steps to reach the goal, containing no more than 3 dead ends, and the length of the optimal path did not exceed 43 units. The moderate difficulty consisted of a maze of 15 × 15 cells, requiring approximately 22 steps, containing 4 dead ends, and the optimal path had a length of about 67 units. The high difficulty was characterized by a maze of 19 × 19 cells, requiring 33 steps to reach the goal, containing 7 dead ends, and the length of the optimal path reached 109 units. These parameters clearly define the navigational challenge and allow for objective evaluation of the impact of maze complexity on users’ navigation abilities in virtual reality. The maze design was refined with input from an expert panel consisting of specialists in virtual reality, cognitive psychology, and logistics, ensuring that the maze structure and complexity were aligned with both theoretical and practical considerations.

The mazes were created in the Unity 3D environment, which enabled efficient management and editing of all maze components. Individual elements, such as walls, floors, and other environmental features, were modeled in Blender software, exported as optimized FBX models, and then imported directly into Unity. Maze generation was performed using the Umbra Maze Magician tool, which facilitated the rapid and repeatable creation of structures with defined complexity and variability. After generating the basic layout, the optimal paths were automatically determined using the Pathfinding Tool, and these were manually verified and adjusted. Finally, custom C# scripts were implemented to provide functionalities such as time tracking, user movement control, teleportation, and interactions with the maze walls. The entire process of creation included repeated optimization to ensure that the final VR application ran smoothly without undesirable technical issues.

The final 3D virtual maze was designed as an abstract model of a warehouse space. The maze consisted of several intersections where participants had to choose between two directions—left or right. To improve orientation and support decision-making, waypoints were strategically placed throughout the maze, resembling objects typical of warehouse environments (e.g., shelves, pallets, or boxes of various sizes). These waypoints acted solely as neutral visual landmarks rather than directional prompts. They were positioned at every T-junction and mid-corridor bend, with identical objects mirrored on alternative branches so that no waypoint singled out the optimal path. Thus, their purpose was to maintain ecological plausibility and aid general orientation without offering explicit hints that could bias maze-solving performance. The complexity of the maze was controlled to ensure comparability of results across trials, with participants tasked to efficiently navigate from the starting point to the defined goal. The environment was designed in neutral colors with minimalist textures, which reduced visual distractions and allowed participants to focus fully on the spatial navigation task. For each difficulty level, three different variants of the VR maze were prepared, which were assigned to participants in random order. The use of a warehouse environment ensures that the results are easily transferable to practical VR applications in logistics, warehouse operations management, or workplace ergonomics optimization.

### Participants

The study included fifteen university students (ten males, five females) aged between 20 and 25 years (M = 22.6, SD = 1.64), recruited on a voluntary basis from technical and humanities disciplines. All participants had normal or corrected-to-normal vision and reported no history of vestibular disorders, epilepsy, or other medical conditions that could compromise their ability to navigate virtual environments. To verify functional stereoscopic vision, each volunteer completed the Randot^®^ Stereotest (Stereo Optical Co., Chicago, IL). All achieved a stereo-acuity threshold of ≤ 60 arc sec (M = 42 ± 12 arc sec), indicating normal stereopsis^41^. Before enrolment, all volunteers completed the Motion Sickness Susceptibility Questionnaire-Short (MSSQ-S). Candidates scoring above the 75th percentile^42^ were excluded to minimise the risk of pronounced cybersickness. The final sample’s mean MSSQ-S score was 19.3 ± 6.4, corresponding to the 42nd percentile, which indicates low-to-moderate baseline susceptibility.

All procedures involving human participants were approved by the Institutional Ethical Committee of University of West Bohemia and conducted in accordance with the Declaration of Helsinki and relevant institutional guidelines and regulations. Written informed consent was obtained from all participants.

To determine the minimum required sample size, an a priori power analysis was conducted using G*Power 3.1.9.4. A repeated-measures ANOVA design, with three experimental conditions (HTR, CTR, CBS), was assumed for the analysis. A medium effect size (f = 0.25), an alpha level of α = 0.05, and a desired power of 80% were used for the calculation. The estimated correlation between repeated measures was set at 0.75, reflecting a high consistency of individual performance across the different VR conditions. The result of the power analysis indicated that a minimum sample size of 15 participants was required. Although our final analyses were non-parametric (Kruskal–Wallis test), we initially based the sample-size estimation on ANOVA, as non-parametric tests generally have similar or slightly lower statistical power^46^.

Participants had varying levels of VR experience: approximately 50% minimal, 25% none, and 25% above average. Experience levels were classified with a brief intake questionnaire: “none” = fewer than five lifetime VR sessions; “minimal” = occasional use (less than once per month during the previous year); “above-average” = at least weekly sessions of one hour or more, or recent involvement in VR content creation within the last six months. Participants also reported their prior exposure to the three locomotion techniques under study. None had previously used Cybershoes; the eight participants with minimal overall VR experience had limited contact with joystick-based locomotion in commercial titles (≤ 10 lifetime sessions), the three with above-average VR experience had tried hand-tracking teleportation once or twice, and the remaining four had no prior exposure to any of the test techniques. To mitigate potential learning effects, participants underwent a standardized familiarization phase before the experimental trials. Testing was conducted in controlled laboratory conditions, with a maximum of three participants present per session. However, each participant performed the trials independently to ensure that navigation performance and subjective assessments were not influenced by external factors. No financial compensation was provided.

### Procedure

Participants were introduced to the study protocol and provided with a detailed explanation of the locomotion interfaces. A short familiarization phase (approximately two minutes per locomotion condition) preceded the main experimental trials to minimize motor learning effects. The two-minute familiarization phase was conducted in a separate practice area outside the experimental mazes, where participants could freely move, turn, and stop with each locomotion interface. No maze-solving tasks were performed and no performance data were recorded during this phase. Each participant completed nine maze navigation trials, divided among three locomotion methods: HTR, VR CTRs, and CBS and three difficulties. This within-subject design ensured that all participants experienced each locomotion method. The mazes were designed to progressively increase in difficulty based on three key parameters: size, path length, and the number of dead ends. During the familiarization phase, participants were introduced to the control methods: HTR, VR CTRs, and CBS. For HTR, participants were guided through the process of controlling movement using hand gestures. The VR CTR condition familiarized participants with traditional joystick-based movement, while the CBS condition involved seated locomotion, where participants learned to perform stepping motions to navigate the virtual environment. Each locomotion method was tested across three difficulty levels (I - easy, II - moderate, III - hard), progressing from the easiest to the most challenging level. The order of testing for the different locomotion methods was randomized across participants using the Excel function RAND. Specifically, each participant received a unique pseudo-random sequence of the three locomotion conditions (HTR, CTR, CBS), so that the starting modality differed across the sample, while the progression through maze difficulties (levels I → III) remained fixed within each modality. The fixed easy-to-hard sequence was chosen to ensure a consistent increase in challenge and to facilitate within-subject comparisons at each difficulty level. Each difficulty level was characterized by a predefined and constant set of parameters (maze size, path length, number of steps, and number of dead ends) to ensure consistent and comparable difficulty across all locomotion methods within each level. Before the familiarisation phase, participants also filled in the MSSQ-S; their scores were recorded for descriptive purposes and were not used to stratify trial order.

Trial order was randomized to eliminate learning effects and the influence of trial sequence on experimental outcomes. However, within each locomotion method (HTR, CTRs, CBS), participants navigated the mazes in the same order of difficulty (from level I to level III). Prior to each trial, participants were allowed a brief rest period (approximately three minutes) to mitigate potential cybersickness symptoms. Each navigation task commenced upon the participant’s virtual entry into the maze and concluded when they reached the designated exit point. An automated timing system recorded completion times, which were verbally confirmed by the participant to the experimenter.

Following the completion of all trials, participants completed a post-experiment questionnaire assessing usability and cybersickness levels. Usability was measured using the System Usability Scale (SUS), while cybersickness intensity was self-reported using a five-point scale ranging from 1 (no discomfort) to 5 (severe discomfort). We selected this concise single-item rating to keep the post-experiment questionnaire brief; administering multi-item instruments such as the 16-item Simulator Sickness Questionnaire (SSQ) or the 9-item Virtual Reality Sickness Questionnaire (VRSQ) for each locomotion condition would have added substantial time and cognitive load. Prior work shows that such Likert-style global ratings correlate strongly with SSQ total scores (*r* ≈ 0.7–0.8) and are therefore considered a valid quick-screening tool for cybersickness^43^. The 16-item Simulator Sickness Questionnaire (SSQ)^44^ was considered, yet pilot testing showed that completing it after each of the nine trials added ≈ 10 min and noticeably increased fatigue. Because our protocol already included the SUS and open-ended questions, we adopted the single-item discomfort rating as a time-efficient substitute; this brief scale has repeatedly demonstrated high correlation with SSQ totals.^4345^ The entire experimental session lasted between 25 and 35 min per participant, including briefing and questionnaire completion. Participants were free to terminate their participation at any time without providing a reason.

### Data analysis

The collected data were analyzed using non-parametric statistical methods due to the relatively small sample size and the non-normal distribution of completion times. The primary dependent variables included maze completion time (in seconds), subjective usability ratings (System Usability Scale, SUS), and self-reported cybersickness levels (five-point scale).

To evaluate differences in maze navigation performance across locomotion methods, a Kruskal–Wallis test was conducted, followed by post hoc pairwise comparisons using Bonferroni correction to account for multiple comparisons. The null hypothesis (H₀) stated that there were no significant differences in maze completion times between locomotion methods, whereas the alternative hypothesis (H₁) proposed that locomotion type significantly influenced navigation efficiency​. The statistical threshold for significance was set at *p* < 0.05.

For assessing differences in cybersickness intensity across conditions, a separate Kruskal–Wallis test was performed. The hypothesis (H₂) suggested that different locomotion techniques induce varying degrees of cybersickness. Subjective cybersickness scores were analyzed to determine whether teleportation (HTR), CTR-based movement, or CBS resulted in significantly different discomfort levels​.

Additionally, to explore potential relationships between navigation performance and subjective experiences, Spearman’s rank correlation coefficient was computed for the following associations: (1) completion time vs. cybersickness intensity, and (2) completion time vs. SUS scores. The analysis aimed to determine whether faster navigation was associated with lower cybersickness or higher usability ratings. The correlation results indicated no statistically significant relationships, with rₛ ≈ 0.22 (*p* = 0.18) for cybersickness and rₛ ≈ − 0.28 (*p* = 0.10) for usability, suggesting weak and non-significant trends​.

All statistical computations were performed using R (version 4.2.2), with supplementary packages for non-parametric testing and visualization.

## Results

The analysis of maze completion times revealed statistically significant differences among the three locomotion methods. A Kruskal–Wallis test confirmed that the type of locomotion had a significant effect on navigation performance (*p* < 0.05). Post hoc Bonferroni-adjusted comparisons demonstrated that participants using HTR with teleportation required significantly more time to complete mazes compared to both CTR-based movement and CBS (*p* < 0.01). The difference between CTR and CBS was significant (*p* < 0.05) in the two higher difficulty levels, while no significant difference was observed in the simplest maze category (*p* > 0.05).

The mean completion times (in seconds) with standard deviations for each locomotion method across difficulty levels are presented in Table 2. The results indicate that HTR consistently resulted in the slowest navigation performance, while CBS exhibited a slight advantage over CTR in more complex navigation tasks.

**Table 2:** Maze completion times (Mean ± SD in seconds) to clearly illustrate performance differences among locomotion methods, direct comparisons between HTR, CTR, and CBS—reporting both the absolute time differences (Δ, in seconds) and the corresponding percentage increases—are presented in Tables 3; Fig. 3. The data confirm that CTRs were significantly faster than HTR across all conditions, while CBS outperformed both methods in higher complexity categories.

Category	HTR (M ± SD)	CTR (M ± SD)	CBS (M ± SD)
I (Easy)	127 ± 54	52 ± 25	52 ± 22
II (Moderate)	93 ± 37	66 ± 29	63 ± 31
III (Hard)	141 ± 58	115 ± 42	108 ± 51

**Table 3:** Comparison of locomotion methods (Mean completion time and absolute Difference).

Comparison	Category I (Easy)	Category II (Moderate)	Category III (Hard)	Faster locomotion
HTR vs. CTRs	127 vs. 52 (144%) | Δ = 75s	93 vs. 66 (41%) | Δ = 27s	141 vs. 115 (23%) | Δ = 26s	CTRs
HTR vs. CBS	127 vs. 52 (144%) | Δ = 75s	93 vs. 63 (48%) | Δ = 30s	141 vs. 108 (31%) | Δ = 33s	CBS
CTRs vs. CBS	52 vs. 52 (0%) | Δ = 0s	66 vs. 63 (5%) | Δ = 3s	115 vs. 108 (6%) | Δ = 7s	CBS

**Fig. 3:**
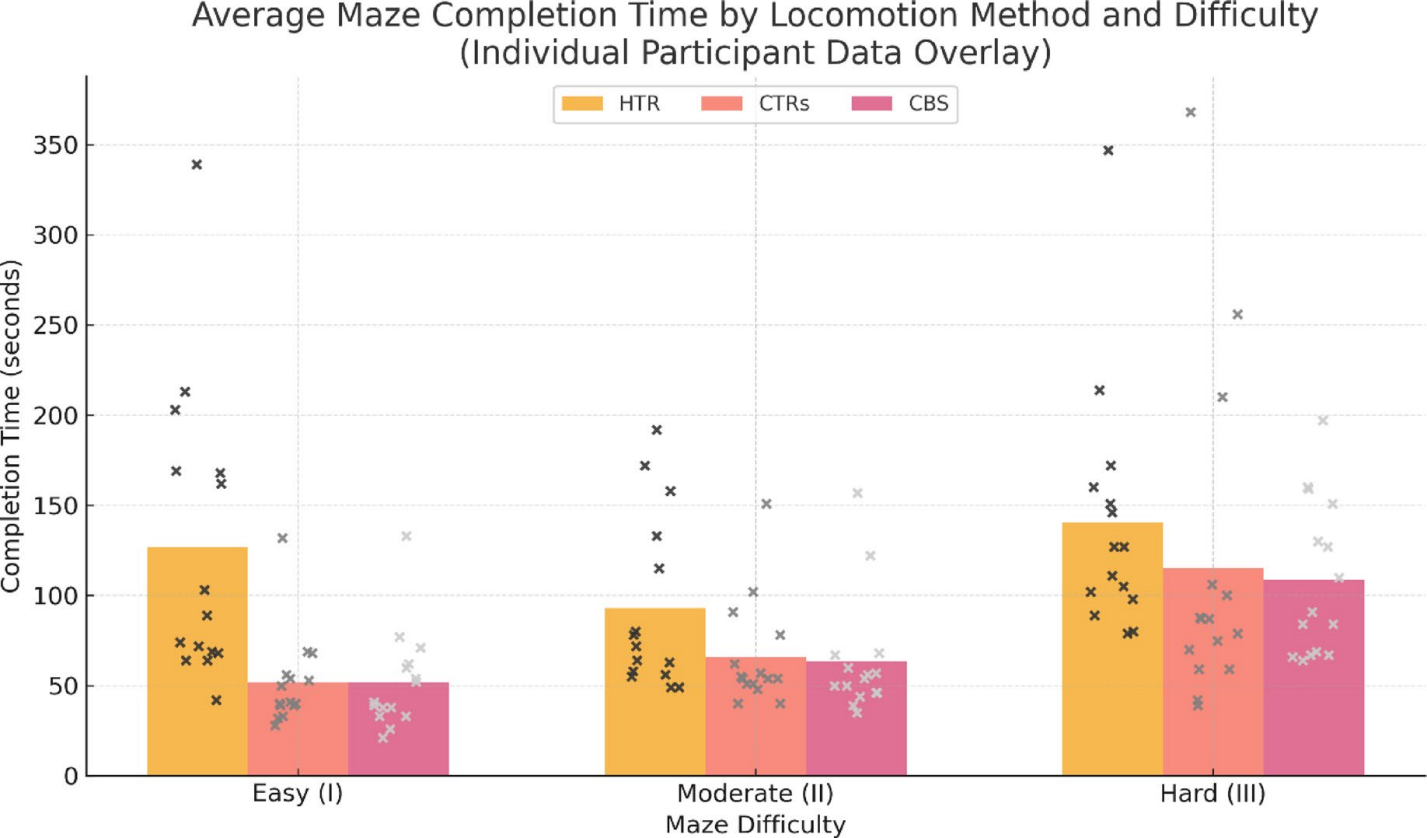
Graphical comparison of Locomotion Methods (Mean Completion Time). Individual completion times (*n* = 15) are super-imposed as jittered grayscale markers on each bar, allowing direct visualisation of the variability within each locomotion method and difficulty level.

**Fig. 4:**
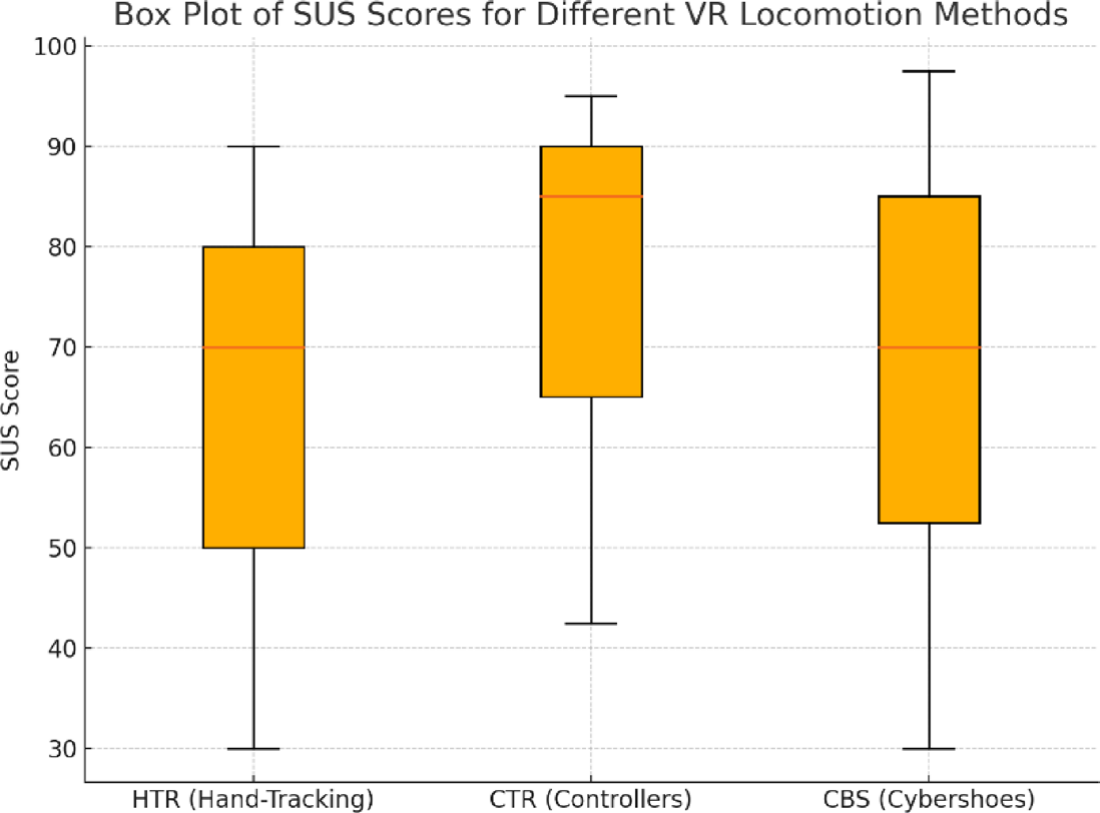
SUS Scores for Different VR Locomotion Methods.

User experience was assessed using the System Usability Scale (SUS). The mean subjective usability ratings are presented in Table 4; Fig. 4. CTR received the highest SUS score, followed by CBS and HTR. A Mann–Whitney test with Bonferroni correction revealed a statistically significant difference between CTR and HTR (*p* < 0.01), indicating that users perceived joystick-based movement as more intuitive and efficient compared to teleportation. The difference between CTR and CBS was on the threshold of statistical significance (*p* = 0.07), with a trend favoring CTR in terms of usability perception.

**Table 4:** Subjective usability ratings (SUS) (Mean ± SD).

Locomotion method	SUS score (M ± SD)
HTR	65.83 ± 22.22
CTR	74.67 ± 18.52
CBS	67.83 ± 24.07

The analysis of cybersickness intensity, measured on a five-point scale (1 = no discomfort, 5 = severe discomfort), indicated significant differences among locomotion methods (*p* < 0.05). The mean cybersickness scores were HTR: 1.8 (SD = 0.9), CTR: 2.3 (SD = 1.1), and CBS: 2.9 (SD = 1.2). Post hoc analysis confirmed that CBS induced significantly higher levels of cybersickness compared to HTR (*p* = 0.006), while CTR occupied an intermediate position without a significant difference from either condition (*p* = 0.12). These results support the second hypothesis (H2), which predicted that different locomotion methods would yield distinct cybersickness levels.

Spearman’s rank correlation was used to explore relationships between navigation performance and subjective experience. The correlation between completion time and cybersickness was weak and non-significant (rₛ = 0.22, *p* = 0.18), suggesting that faster navigation was not directly associated with lower cybersickness. Similarly, the correlation between completion time and usability (SUS scores) was also non-significant (rₛ = −0.28, *p* = 0.10), indicating that faster maze completion did not consistently correspond to higher usability ratings.

The results indicate that the choice of locomotion method significantly influences spatial navigation performance and user experience in VR. HTR was consistently the slowest method, while CBS demonstrated a slight performance advantage over CTR in more complex mazes, though this difference was not statistically robust across all conditions. These findings support H1, confirming that locomotion techniques significantly affect navigation efficiency, leading to the rejection of the null hypothesis (H0), which assumed no differences in completion times.

The results also confirm that different locomotion methods lead to distinct levels of cybersickness, with CBS inducing significantly higher cybersickness compared to HTR (*p* = 0.006), while CTR occupied an intermediate position without a statistically significant difference from either condition (*p* = 0.12). This supports H2, demonstrating that VR locomotion techniques have a measurable impact on cybersickness levels, leading to the rejection of the corresponding null hypothesis (H0).

However, the analysis revealed no clear correlation between navigation performance and subjective experience metrics. The weak and statistically insignificant associations (rₛ = 0.22, *p* = 0.18 for cybersickness and rₛ = −0.28, *p* = 0.10 for usability) suggest that faster or slower movement through the virtual environment does not necessarily correspond to greater or lesser discomfort or perceived usability. This finding refines the interpretation of RQ2, indicating that subjective experience in VR is influenced by multiple factors beyond navigation efficiency alone.

These results highlight the need for further research to examine individual differences in VR motion sensitivity and broader variations in user perception. A larger and more diverse participant sample may help clarify the complex relationship between locomotion efficiency and user comfort, contributing to a more refined understanding of the trade-offs in VR navigation design. Exploratory subgroup analyses examined whether prior VR-experience level (none *n* = 4, minimal *n* = 8, above-average *n* = 3) modulated outcomes. A Kruskal–Wallis test showed no significant main effect of experience on maze-completion time (H = 1.08, *p* = 0.58) or on cybersickness ratings (H = 1.97, *p* = 0.38). Median completion times in the hard maze were 123 s (none), 118 s (minimal), and 114 s (above-average), while median CBS discomfort ratings were 3.2, 3.4, and 3.7 on the five-point scale, respectively. Thus, although the most experienced users finished slightly faster, they still reported the highest discomfort with Cybershoes. Given the small, imbalanced sub-samples, these patterns should be interpreted with caution.

## Discussion

Grounded in Sensory Conflict Theory, the present results show that differences in visual-vestibular-proprioceptive mismatch across locomotion modes predicted both performance and discomfort. Teleportation created the smallest conflict and therefore the least cybersickness, but the absence of continuous optic flow impeded path integration and slowed navigation. Joystick steering maximised visual–vestibular mismatch yet preserved continuous flow cues, yielding high efficiency at the cost of moderate discomfort. Cybershoes re-introduced lower-limb proprioception, narrowing the mismatch and boosting speed in complex mazes, but the seated posture left vestibular cues absent, explaining the residual sickness. These patterns accord with contemporary extensions of Sensory Conflict Theory that emphasise proprioceptive weighting^10,11^ and with evidence that higher refresh rates can attenuate—but not eliminate—conflict-driven sickness^37^. Our findings therefore provide empirical support for the theory while extending it to seated walking-in-place devices.

This study investigated how different virtual reality locomotion methods influence spatial navigation performance, usability, and cybersickness. The findings confirm that locomotion modality significantly impacts task efficiency and subjective user experience, with each method presenting distinct advantages and limitations. This aligns with broader research on Virtual Locomotion Techniques (VLTs), which emphasizes that different methods involve trade-offs between immersion, control precision, and cybersickness mitigation. Prior studies further indicate that no single locomotion technique is universally optimal, as each imposes unique cognitive and physiological demands on users^46^.

The prolonged maze completion times observed for HTR with teleportation corroborate existing research indicating that discontinuous locomotion disrupts cognitive mapping and spatial orientation. The lack of continuous movement cues likely necessitates reliance on discrete positional updates rather than the formation of a cohesive spatial representation, thereby increasing cognitive load and response latency. Bowman and McMahan (2007)^47^highlight that discontinuous locomotion, such as teleportation, hinders the development of an internalized spatial model, as users are deprived of natural motion cues essential for wayfinding. Studies on teleportation-based navigation suggest that, while this method effectively mitigates cybersickness by eliminating optic flow, it concurrently diminishes the user’s ability to accurately estimate distances and maintain spatial awareness^37,29^. Boletsis and Cedergren (2019)^48^ further support this assertion, noting that teleportation, despite its usability advantages, can reduce spatial presence due to the lack of continuous movement cues, which may impact user immersion and spatial awareness. The significantly lower cybersickness levels recorded for HTR are consistent with these findings, reinforcing teleportation as a viable solution for individuals susceptible to motion sickness, albeit at the expense of navigation efficiency^49^.

The CTR-based locomotion method exhibited an optimal balance between navigational efficiency, usability, and motion comfort. The elevated usability scores suggest that participants perceived joystick-based navigation as both intuitive and effective, corroborating prior findings that CTRs facilitate precise modulation of speed and directional control, thereby reducing cognitive load in navigation tasks^50^. This is consistent with existing research indicating that joystick locomotion constitutes an established interaction paradigm, widely recognized by users familiar with gaming interfaces, and provides robust technical performance with minimal tracking inconsistencies^51^. By manipulating the joystick, users can dynamically adjust their viewpoint and navigate in any direction, allowing for flexible and responsive control over movement^6^. Although cybersickness scores were higher relative to teleportation, the absence of statistically significant differences between conditions suggests that moderate exposure to joystick-based locomotion does not inherently provoke severe discomfort. Previous studies have highlighted that prolonged engagement with joystick locomotion may pose challenges due to the sustained optic flow it generates, which lacks congruence with vestibular cues inherent to natural walking^6^. In contrast, Langbehn et al. (2018)^30^ reported that participants exhibited a preference for teleportation over joystick-based locomotion, associating it with a reduced incidence of motion sickness. Additionally, some studies have noted that prolonged use of hand-held VR CTRs may introduce physical strain on specific musculoskeletal regions due to the necessity of continuous manual engagement, potentially leading to fatigue and discomfort over extended durations^8^. Within the temporal constraints of this study, CTR proved to be an efficient and pragmatically viable navigation method. Our findings align with this, as CBS, while providing a more immersive experience, led to higher levels of cybersickness compared to HTR. In contrast, HTR with teleportation minimized physical strain but resulted in slower navigation times and reduced spatial orientation, highlighting the trade-offs between comfort and navigation efficiency in different locomotion methods.

CBS demonstrated navigation performance comparable to traditional CTRs, slightly exceeding CTR performance in more complex mazes. This outcome aligns with the premise that engaging the lower limbs, even within a seated posture, may enhance proprioceptive feedback and contribute to improved spatial orientation^52^. The slight performance advantage observed in higher-difficulty mazes suggests that locomotion strategies emulating natural gait dynamics may facilitate a more robust internalized spatial representation. However, despite these potential advantages, CBS were associated with the highest levels of cybersickness, a finding that contradicts initial hypotheses suggesting that semi-natural walking simulations might alleviate motion discomfort. These findings align with vestibular-visual conflict theory, which predicts peak cybersickness when optic-flow acceleration is decoupled from both vestibular and proprioceptive inputs^10^. In CBS this mismatch is intensified by the seated posture, and laboratory work shows that greater postural instability while viewing optic flow reliably precedes visually induced motion sickness^53^. Optimising CBS should therefore focus on tightening the temporal coupling between foot-sliding kinematics and optic-flow velocity and on adding auxiliary cues—such as subtle seat tilt or vibrotactile footplates—that restore synchrony between lower-limb proprioception and perceived self-motion. Interfaces that achieve sub-50 ms synchrony between gait phase and visual update already report 20–30% lower symptom scores despite similar visual-flow speeds^12^, suggesting a viable direction for future CBS design. In addition, any temporal or spatial mismatch between the foot-stepping input and the corresponding visual flow can further amplify sensory conflict, as even small delays or scaling inaccuracies in mapping foot kinematics to optic-flow velocity increase cybersickness severity. Ensuring high-fidelity synchronization and calibration of stepping motions to visual feedback is therefore essential for reducing discomfort in seated walking-in-place interfaces.

While previous studies have suggested that semi-natural locomotion techniques may attenuate cybersickness by minimizing sensory conflict^12^ further research has demonstrated that body posture significantly influences the severity of cybersickness symptoms, indicating that seated and standing positions can differentially impact user comfort and susceptibility to motion sickness^34^. The present findings indicate that the seated posture required for CBS may have disrupted the natural coordination between lower-limb movement and vestibular feedback, resulting in a sensory incongruence that exacerbated cybersickness symptoms. Similar patterns have been documented in other semi-natural locomotion systems, particularly in cases where movement mechanics fail to align seamlessly with real-world ambulatory dynamics^34,54^. These findings suggest that further refinement of the CBS system may be necessary, such as enhancing the synchronization between stepping motion and visual feedback or incorporating additional stabilization mechanisms to mitigate vestibular inconsistencies.

The absence of a significant correlation between navigation speed and cybersickness suggests that locomotion efficiency and user comfort depend on distinct underlying factors. Although it is commonly assumed that reduced movement velocity mitigates cybersickness^9^ the present findings suggest that individual susceptibility exerts a more pronounced influence than absolute navigation speed. Prior studies have shown that proficient navigation performance does not necessarily prevent simulator sickness. Conversely, individuals who navigate less efficiently may experience minimal cybersickness symptoms. User characteristics introduce an additional layer of complexity in understanding the interplay between hardware, content, and VR-induced discomfort, further highlighting the multifaceted nature of cybersickness^45,39^. Similarly, the weak correlation between usability ratings and navigation efficiency implies that subjective perceptions of ease of use do not necessarily correspond to enhanced movement fluency. While some participants commended CTRs for their “comfortable operation,” others expressed a preference for the “natural feel” of CBS despite experiencing mild physical discomfort. These findings underscore the multifaceted nature of VR locomotion, wherein complex interactions between cognitive and physiological mechanisms shape the overall user experience.

While these findings offer valuable insights, several limitations should be acknowledged. The small sample size (*n* = 15) restricts the generalizability of the results, as individual differences in VR experience, cybersickness susceptibility, and spatial cognition may have influenced the outcomes. In addition, although participants with high MSSQ-S scores were excluded, the modest sample prevented us from analysing susceptibility as a covariate. A larger, more diverse sample would enhance statistical power and allow for a deeper analysis of variability among users. Moreover, the unequal and modest experience sub-groups (4/8/3) limited statistical power; the exploratory tests detected no reliable experience-related effects, so larger balanced samples are required to confirm whether prior VR familiarity moderates performance or cybersickness.

Second, the locomotion methods were not tested on identical head-mounted displays. The Cybershoes trials used the tethered Valve Index (1440 × 1600 px per eye, 144 Hz), whereas the HTR and CTR trials ran on the standalone Quest 2 (1832 × 1920 px per eye, 72 Hz). Hardware differences such as refresh rate and angular resolution can affect both task performance and cybersickness; higher refresh rates, for instance, tend to enhance presence but may also intensify motion-induced discomfort^3,37^. Consequently, part of the elevated cybersickness observed with CBS might stem from headset characteristics rather than the locomotion interface itself. Future studies should counterbalance locomotion methods across identical HMDs—or statistically control for display parameters—to isolate the effect of locomotion modality however, display-parameter differences between the Quest 2 and Valve Index could have biased our results, in particular because the Index’s higher refresh rate may have mitigated cybersickness or altered task performance in the CBS condition.

Furthermore, although maze complexity was validated by objective parameters and increasing completion times, we did not administer a dedicated workload inventory such as the NASA Task Load Index (NASA-TLX)^55^. Incorporating NASA-TLX in future studies would provide convergent evidence that the three difficulty levels differ not only behaviourally but also in perceived cognitive load.

We also acknowledge that presenting maze levels in a fixed order may have introduced practice effects; future studies could employ a counterbalanced or Latin-square ordering of difficulty to mitigate such learning effects.

Additionally, the study focused on a single VR task—maze navigation—whereas other interactions, such as object manipulation or social VR exploration, may yield different usability and comfort dynamics. The relatively short experimental sessions further limit the ability to assess long-term adaptation effects, which may influence both navigation efficiency and cybersickness levels over extended use. Environmental factors, including field-of-view settings, locomotion acceleration, and individual control preferences, were not systematically varied but could significantly impact performance and comfort. Future research should explore these aspects to develop a more comprehensive framework for optimizing VR locomotion. Beyond total completion time, future studies should also log granular navigation metrics—such as the number of user inputs, wrong turns, and dead ends encountered—to better characterise spatial strategies and the cognitive mapping demands of each locomotion interface.

## Conclusion

This study examined the impact of different VR locomotion techniques on spatial navigation efficiency, usability, and cybersickness in virtual maze environments. The findings confirm that locomotion modality significantly influences both objective performance metrics and subjective user experience. Teleportation, while minimizing cybersickness, was associated with the slowest navigation times, likely due to its disruptive effect on spatial awareness and cognitive mapping. CTR-based locomotion provided a well-balanced solution, offering precise and efficient movement without excessive motion discomfort. CBS, despite enabling a more natural stepping motion, induced the highest levels of cybersickness, suggesting that semi-natural locomotion techniques may require further refinement to optimize user comfort and performance.

Although the results clearly define strengths and weaknesses of each locomotion method, future research could incorporate detailed measurements of spatial cognition and physiological indicators for a more comprehensive understanding of user experience. The analysis of adaptation effects over prolonged VR use would help refine the optimal locomotion method for specific applications and user types. Exploring hybrid locomotion techniques, such as integrating CBS with intermittent teleportation or dynamically adjusting movement speed based on real-time cybersickness monitoring, may provide adaptive solutions for balancing user comfort and performance. Optimizing environmental factors could help mitigate cybersickness. Examples include adjusting the field-of-view and employing gradual acceleration techniques.

From an applied perspective, these results inform the selection of locomotion methods based on specific VR use cases. Teleportation remains the preferred choice for applications prioritizing motion comfort over navigational efficiency, such as exposure therapy or casual exploration. Joystick-based locomotion is optimal for tasks requiring speed and precision, including industrial simulations and virtual prototyping. CBS, while offering a more embodied interaction, may benefit from improved foot-tracking algorithms or enhanced vestibular feedback mechanisms to mitigate cybersickness and enhance spatial presence.

## Data Availability

The datasets generated and analyzed during the current study are available from the corresponding author upon request.
